# Remaking the Self: Trauma, Teachable Moments, and the Biopolitics of Cancer Survivorship

**DOI:** 10.1007/s11013-012-9276-9

**Published:** 2012-10-09

**Authors:** Kirsten Bell

**Affiliations:** Department of Anthropology, University of British Columbia, 6303 NW Marine Dr, Vancouver, BC V6T 1Z3 Canada

**Keywords:** Cancer, Trauma, Lifestyle, Development, Psychosocial oncology

## Abstract

As numerous scholars have noted, cancer survivorship is often represented in popular discourse as providing an opportunity for a physical, emotional, and spiritual makeover. However, this idea that cancer enables the self to be remade on all levels is also increasingly evoked in the field of psychosocial oncology. Exploring cancer survivorship as a biopolitical phenomenon, I focus on two concepts that have become central to understandings of the disease: the “teachable moment” and “post-traumatic growth.” Drawing primarily on representations of cancer survivorship in the clinical literature, I suggest that cancer is increasingly seen to present a unique opportunity to catalyze the patient’s physical and psychological development. In this framework, the patient can no longer be relied upon to transform him or herself: this change must be externally driven, with clinicians taking advantage of the trauma that cancer entails to kick-start the patient into action. Broadening my analysis to the concepts of “trauma” and “development” writ large, I go on to suggest that survivorship discourse seems to partake of a larger and relatively recent meta-narrative about development—both individual and societal—and the positive opportunity that trauma is seen to present to stimulate reconstruction on a grand scale.

A survivor is a triumphant person who lives with, after, or in spite of a diagnosis or traumatic event. Survivors refuse to assume the identity of their adversity. They are not imprisoned by the constructs of a label. Instead, survivors use their brush with mortality as a catalyst for creating a better self. We transform our experience in order to further evolve spiritually, emotionally, physically, and mentally.

(Carr 2008)

## Introduction

In June of 2010, the biennial *Cancer Survivorship Research: Recovery and Beyond* conference was held in Washington DC. Sponsored by the National Cancer Institute, the American Cancer Society, LIVESTRONG: the Lance Armstrong Foundation, and the Centers for Disease Control and Prevention, the meeting is devoted exclusively to the topic of cancer survivorship and its stated aim is to “bring together investigators, clinicians, and survivors to share and learn about the most up-to-date cancer survivorship research” (NCI, ACS, LIVESTRONG, CDC 2010).

The first day of the 2010 conference was devoted to the relationship between lifestyle and cancer and the line up of presenters included an impressive list of “leading lights” in the field. Despite the generally inconclusive state of the evidence regarding the impact of diet, weight, and exercise on cancer survival, the message broadcast to participants was of the need to actively encourage patients to modify their lifestyles (see Bell 2010; Bell and Ristovski-Slijepcevic under review). “We need to encourage survivors to increase their physical activity after diagnosis” stressed one well-known dietitian. A second reiterated the need to “capitalize on the teachable moment caused by the cancer diagnosis” in promoting weight loss among overweight cancer patients. In her closing remarks, the moderator of the final session of the day tasked the audience with spreading this message far and wide. “Talk about exercise to the survivors you come into contact with,” she urged. “We’re charging you to get the message out”: “spread the word.”

The remainder of the conference was more varied in content and included sessions on topics such as rehabilitation, sexuality, fertility, and the psychological impacts of cancer survivorship. *Benefit Finding and Growth After Treatment for Cancer* was one of the most popular panels, unsurprising given the growing prominence of these concepts in the field of psychosocial oncology over the past 15 years. The session focused on describing and measuring “benefit finding” and “post-traumatic growth” in cancer survivors, and the permanency of their effects over time.^1^ Informing several of the presentations was the assumption that there is a “right” way to deal with cancer, a position increasingly echoed in the psychosocial literature. In the words of one speaker: “if we respond [to cancer] in a healthy way we can find something out of it,” and his talk ended with a discussion of recent efforts to develop interventions to “facilitate” post-traumatic growth in cancer survivors. Significantly, the key message of the conference was that *of* intervention: the importance of intervening in the lives of cancer survivors, in a cost effective way, to make them thinner, fitter, and psychologically and spiritually healthier.

In 1977 Susan Sontag (1990) wrote a searing indictment of the prevailing cultural view of cancer as a “death sentence.” However, in the 35 years since the publication of her essay, the equation of cancer and death has been at least partially superseded by the assertion that cancer “can be beaten” (Sinding and Gray 2005; Deimling et al. 2007). These developments speak to the emergence of a new discourse on cancer, a discourse centered on survival rather than death, which has resulted in the production of a new category of person: the “cancer survivor” (Saillant 1990; Zebrack 2000).

Understood as a distinct clinical entity, “cancer survivors” are the site of considerable media interest and medical attention. If cancer was unspeakable only a few decades ago, today it has become a disease to publicly lay claim to and even celebrate. While this discursive shift can be seen as positive in so far as it moves the emphasis away from the cancer “victim” identity that Sontag railed against, there is also a downside to the emergence of discourses on cancer survivorship (Deimling et al. 2007). As a growing number of observers have noted, central to the “cancer survivor” identity is the idea that cancer creates a better self. Barbara Ehrenreich (2001, 2009), writing about breast cancer, has noted that cancer is increasingly conceptualized as a “harbinger of personal growth” and a “makeover opportunity.” In this discourse, which Ehrenreich suggests has become hegemonic, the breast cancer survivor is expected to emerge from the cocoon of cancer diagnosis and treatment as a new and improved person. Expanding on this insight, Sinding and Gray (2005) have identified “spunky survivorship” as the dominant discourse on breast cancer, with survival represented as both an accomplishment and opportunity for self-transformation. Kaiser (2008) has similarly highlighted the popular emphasis on the “exceptionality” of breast cancer survivors.

Although some analyses have suggested that these representations are gendered, with women more often depicted as transformed by the experience of cancer^2^ than men (Seale 2002), it is clear that the “new and improved cancer survivor” trope extends well beyond the realm of breast cancer and pervades popular representations of cancer survivorship more broadly. Today, narratives of personal transformation and self-improvement have become ubiquitous, featuring stories of “heroic struggle and psychological progress in the face of the disease” (Seale 2001, p. 428; see also Stacey 1997; Little et al. 2002). For example, in their analysis of print news coverage of cancer survivorship, Kromm, Smith, and Singer (2008, p. 3) note that the disease is depicted in “overwhelmingly positive terms,” with survivors represented as “energized, dynamic survivor warriors.” Lance Armstrong (2006) epitomizes the energized survivor warrior, having famously noted that:

[P]eople have asked me what I mean when I say that given a choice between cancer and winning the Tour de France, I’d choose the cancer. What I mean is that I wouldn’t have learned all I did if I hadn’t had to contend with the cancer. I couldn’t have won even one Tour without my fight, because of what it taught me. I truly believe that (p. 284).

Implicit within such representations is the idea that cancer enables the self to be remade on all levels—psychological, spiritual, *and* physical—a view that has crystallized in recent conceptualizations of cancer survivorship, such as the quote this essay opened with from Kris Carr, the author of *Crazy, Sexy Cancer Survivor*. Yet, as the *Cancer Survivorship Research* conference illustrates, this trope is not restricted merely to popular representations of cancer but infuses oncological discourses as well. As Delvecchio Good et al. (1990, pp. 55–56) have previously reminded us, “A nation’s practice of oncology is shaped not only by medical technology and therapeutics, but by local popular and medical cultures as well.”

This paper critically examines the widespread perception that cancer enables and facilitates self-transformation. Through an exploration of the concepts of the “teachable moment” and “post-traumatic growth” and their embrace in the field of psychosocial oncology, I examine the growing push to intervene in the lives of cancer survivors to “enhance” their lifestyles and psychological development. In the second half of the paper, I broaden the scope to consider the concepts of “trauma” and “development” writ large, before turning to a consideration of the impacts of these discourses on cancer survivors themselves. My overarching goal is to demonstrate that discourses on cancer survivorship are linked with much larger cultural shifts in the ways that psychological, social, and societal development are conceptualized.

## Cancer and the Teachable Moment

In recent years, the concept of the “teachable moment” has become a cornerstone of the health literature to describe naturally occurring life transitions or health events (e.g., cancer diagnosis, hospitalization, pregnancy) that have the potential to motivate individuals to adopt health-protective behaviors (McBride et al. 2003; Ganz 2005). However, the widespread embrace of the concept has not been driven by empirical research demonstrating its validity, which is notably lacking (Lawson and Flocke 2009). Rather, it is generally treated as a self-evident truism, suggesting that much of its appeal stems from an underlying model of human nature and selfhood that appears to intuitively make sense to oncology care professionals.

This underlying model of human nature crystallizes in attempts to describe the elements constituting the teachable moment. Here, the work of McBride et al. (2003) is instructive. They suggest that for cueing events to become teachable moments, three interrelated elements are necessary. The event should (1) increase perceptions of personal risk and outcome expectancies, (2) prompt a strong affective or emotional response, and (3) redefine self-concept or social role. In other words, the concept of the teachable moment suggests that the more scared a patient is, the more fundamentally their prior sense of self is undermined, the higher their motivation is to change their lifestyle—as long as they perceive the change to have some potential benefit.^3^ That fear is central to this model becomes explicit in statements such as “Negative affect, i.e. fear, may be particularly impactful because it increases vigilant attention and prompts the survival instinct” (McBride et al. 2003, p. 163).

It was initially thought that people diagnosed with or “at risk” for cancer would naturally improve their lifestyle because of the exemplary “teachable moment” such events present; however, clinicians are increasingly of the view that the diagnosis of cancer alone cannot be relied upon to stimulate behavior change. In the words of Demark-Wahnefried et al. (2006, p. 5126):

Until recently, there was a degree of optimism among oncologists and researchers because descriptive studies suggested that individuals improved their lifestyle behaviors after being diagnosed with cancer…. [M]ore recent, robust research… reveals that there may be comparatively fewer lifestyle differences between individuals with or without a cancer history than previously thought, especially among long-term cancer survivors. In fact, in some survivor subgroups, the practice of healthful behaviors may even be worse. Thus, although adjusted analyses indicate that survivors may be somewhat more likely to meet physical activity guidelines, the majority are much like the population at large—a population marked by sedentary behavior; overweight or obesity; and suboptimal fruit, vegetable, and fiber consumptions, and high intakes of saturated fat.

Here, the attitudes of those who resist injunctions to modify their lifestyle behaviors are pathologized, deemed to be “marked” by fatalistic attitudes and misconceptions that need to be dispelled by clinicians. As Martha Balshem (1993, p. 67) observes, “The magic bullet is the health-education message, delivered to the target population through an appropriate strategy, preferably at a teachable moment.”

In this framing, teachable moments can be actively *created* and *exploited* rather than simply waited for (Lawson and Flocke 2009, p. 27). Consequently, as the *Cancer Survivorship Research* conference attests, among oncology care providers there has been a growing interest in ways to facilitate and exploit the teachable moment that cancer presents via active interventions that promote adherence to recommended lifestyle behavioral guidelines (Blanchard, Courneya and Stein 2008). According to Demark-Wahnefried et al. (2005, p. 5827):

For decades the cancer diagnosis has been acknowledged as a life-changing event. It is time for oncology care providers to not only lead their patients away from disease but also to capitalize on the teachable moment that cancer provides and guide their patients to better health.

Others similarly argue that formal interventions should be introduced to “take advantage” of the “underused” “window of opportunity” presented by cancer diagnosis and treatment and patients’ “heightened receptivity” during this period (see McBride et al. 2003, 2008; Gritz et al. 2005).

## Trauma and Growth

Underlying these discussions of the teachable moment is the assumption that a cancer diagnosis is deeply traumatizing for those who experience it.^4^ Indeed, the use of the term “survivor” to refer to people diagnosed with cancer shows how central trauma is to conceptions of the disease, given that the label initially described those who had experienced traumas such as attempted genocide, war, natural disasters, or rape. In contemporary formulations of the teachable moment, the trauma of the cancer diagnosis is deemed to have positive utility in transforming the cancer patient’s lifestyle.

The positive role of trauma is articulated even more explicitly in the concept of post-*traumatic* growth, which suggests that trauma may lead to “a greater appreciation of life and changed sense of priorities; warmer, more intimate relationships with others; a greater sense of personal strength; recognition of new possibilities or paths for one’s life; and spiritual development” (Tedeschi and Calhoun 2004, p. 6). Coined in the 1990s, “post-traumatic growth” was explicitly conceptualized as the antonym of “post-traumatic stress,” representing the upside of trauma: the silver lining to its dark cloud^5^ (Tedeschi, Park and Calhoun 1998). Although the concept did not relate specifically to cancer in its initial formulation, it has certainly flourished in the fertile soil of psychosocial oncology and over the past decade it has gained increasing prominence in the field.

In many respects, post-traumatic growth is the psychological counterpart to the teachable moment, whereby the psychological self is transformed along with the physical self. This becomes evident in discussions of the conceptual underpinnings of post-traumatic growth, which is seen to result from a “seismic event” that “shatters an individual’s assumption system, forcing reconfiguration of their schemata” (Aldwin and Levenson 2004, p. 19). Compare this with McBride et al.’s (2003) description of the teachable moment as a “cueing event” that prompts a “strong emotional response” and “redefines self-concept.”

Indeed, in some conceptualizations, the teachable moment collapses into post-traumatic growth, with increases in health-promoting behaviors seen to be an integral manifestation of such growth (see Lechner and Antoni 2004). Moreover, like the teachable moment, post-traumatic growth is posited to happen spontaneously, but it is also something that can purportedly be triggered through interventions that “take advantage of the trauma-induced disruption to the person’s life” (Lechner and Antoni 2004, p. 35; Garland et al. 2007, p. 950). In both of these framings, trauma has an instrumental role to play in remaking the self. By forcing the cancer patient to re-evaluate his or her life, and by shattering taken-for-granted assumptions about the self, trauma allows the self to be remade from the ground up.

However, as Alan Young (1995) and Didier Fassin and Richard Rechtman (2008) have shown, although the concept of trauma is understood to describe a natural and universal phenomenon, it represents a very particular way of thinking about the self. In this conceptualization, trauma *does* something to the self: either the self becomes “stuck” and unable to progress (as in post-traumatic stress disorder) or trauma “kick-starts” the self, positively transforming it (as in post-traumatic growth). This is evident in some of the language used to talk about cancer survivors, where those who have not experienced growth are deemed to be “stuck”^6^ in earlier phases of the trajectory and unable (or unwilling) to move toward the “light” at the end of the tunnel (e.g., Utley 1999; Taylor 2000). Clearly, in this conceptualization, a particular manifestation of “growth” is naturalized.

In this framework, trauma thus gives grounds for the intervention of psychologists and psychiatrists (Fassin and Rechtman 2008, p. 276). Indeed, it could be argued that the psychic trauma that cancer is seen to entail was central to the legitimization of psychosocial oncology as a distinct sub-specialty in the 1990s.^7^ Thus, in an editorial published in the inaugural issue of the journal *Psycho*-*Oncology*, the emotional response of patients and their families to cancer was identified as a central concern of the subfield, with “controlled trials of psychotherapeutic, behavioral and psychopharmacologic interventions” deemed to be one of its primary goals (Holland 1992, p. 6).

Today, one of the most influential of such interventions is “Cancer Transitions: Moving Beyond Treatment,” which was created in 2006 by the Cancer Support Community and LIVESTRONG (both community-based organizations), although it is increasingly being offered at a number of cancer treatment centers in the USA and Canada.^8^ The program is designed to help cancer survivors “redefine how we live our lives from this point forward,” and to “support and empower survivors as they transition from active treatment to post-treatment” (Cancer Support Community 2011). Running over 6 weeks, the program aims to encourage cancer survivors to “take charge” of their survivorship through information and training in a variety of areas, including exercise, emotional health and well being, nutrition, and how to manage their follow up medical care.

There is considerable demand for such programs among cancer survivors, many of whom feel abandoned following the completion of treatment and are looking for ways to maximize their chances of avoiding disease recurrence (Magee and Scalzo 2006). However, through such interventions and programs cancer survivors are also recruited into their responsibilities as “good” biological citizens and therapeutic subjects^9^ (Rose 2007). This obligation becomes quite explicit in the “patient active” concept, which lies at the heart of the Cancer Transitions program. This concept states that “Patients who participate in their fight for recovery along with their healthcare team, *rather than acting as hopeless, helpless, passive victims of the illness*, will improve the quality of their lives and may enhance the possibility of recovery”^10^ (Golant and Thiboldeaux 2010, p. 474, emphasis added).

There is an underlying neoliberal logic to this framing, whereby the cancer survivor is called upon to enter into the process of his or her own self-governance through “endless self-examination, self-care and self improvement” (Petersen 1996, pp. 48–49; see also Rose 1999). Contemporary oncological discourses on cancer survivorship appear to be premised on this neoliberal logic of privatized risk management, whereby the “good” subject/citizen is expected to take responsibility to manage his or her risks of cancer recurrence, to alleviate the financial burden otherwise imposed upon the tertiary healthcare system.

However, what I want to particularly emphasize about such conceptualizations of cancer survivorship is that the radical biographical disruption and discontinuity (Bury 1982; Little et al. 2002) caused by the disease is increasingly seen to present a unique *opportunity* to catalyze the patient’s physical, psychological, and spiritual development. In this framework, the patient can no longer be relied upon to transform him or herself—this development must be externally driven, with clinicians taking advantage of the “trauma-induced disruption” to the person’s life.

## Trauma, Intervention, and Development

At the heart of the trauma narrative detailed above is a story of individual development: of development forestalled or development enhanced. Thus, although development assumes a progressive form of temporality, which trauma clearly disrupts (Burman 2008), I would argue that trauma is increasingly being understood as a precipitant *to* development. Moreover, in my view, this perceived relationship between trauma and development resonates well beyond the field of psychosocial oncology and permeates contemporary politics and biopolitics more broadly. Following Fassin and Rechtman (2008, p. 22), I would, therefore, like to consider how thinking about experience in terms of “trauma” transforms our understanding of human development on a collective as well as individual level.

As noted above, the concepts of “trauma” and the “survivor” bring radically different groups, such as rape victims, cancer patients, war veterans, survivors of the Holocaust, and major natural disasters, into relation. Thus, under contemporary conceptions of trauma,

There is no difference between the survivor of genocide and the survivor of rape… the notion of ‘trauma’ has become a general way of expressing the suffering of contemporary society, whether the events it derives from are *individual* (rape, torture, illness) or *collective* (genocide, war, disaster) (Fassin and Rechtman 2008, pp. 19–20, emphasis added).

Bearing in mind Erica Burman’s (2008) note of caution that it is “as problematic to attempt to read social processes back on to the development of the individual as it is to treat individual development as the prototype and site of manipulation for social development” (p. 9), there appear to be important links between the concepts of “trauma” and “development” writ large that warrant further examination.

The concept of development implies, by definition, potential growth (Castañeda 2002) and is generally used to denote either a state or a process associated with material wellbeing, progress, social justice, economic growth, and personal blossoming (Rist 2006). As Burman (2008, p. 1) notes, “claims to the term ‘development’ inextricably link psychological, cultural and international (social and economic) models and practices.” Thus, models of individual and international development share central key features, assuming that change occurs according to a pre-established pattern, the logic and direction of which is known (Pieterse 1991; Castañeda 2002).

Others before me have explored in detail the shared features of models of individual and international development (e.g., Castañeda 2002; Burman 2008), so I will not repeat their arguments here. However, like models of psychosocial development, models of international development are based on the notion of progression toward a clearly defined endpoint—in this case, a transformed society that shares central features of capitalist modernity (Escobar 1995; Duffield 2001a, 2001b); according to an early iteration of the concept, “ancient philosophies have to be scrapped; old social institutions have to disintegrate; bonds of cast, creed and race have to burst” (quoted in Escobar 1995, p. 4). As Burman (2008, p. 10) notes, stage-based models of international development “not only institute culturally specific norms as if they were universally applicable but also open up strategies for manipulation and intervention.” For my present purposes, it is these strategies that are of particular interest.

Mark Duffield (2001a) suggests that until the mid-1990s the transformational aim of official development policy was generally regarded as something that would emerge naturally through supporting economic growth. In other words, the dominant view was that economic growth would inevitably lead to social change in a desirable direction: i.e., an open market economy, liberal democratic political system, and the breakdown of “backward” forms of custom and practice. However, in the 1990s a new development paradigm emerged which radically altered the meaning of the concept. Key to this “new” conception of development was the idea that social change could “no longer be left to the hoped-for synergies of modernizing projects and market reform” (Duffield 2001a, p. 39). Instead, effecting social transformation became a direct and explicit policy goal. This more aggressively interventionist model of development linked aid policy to conflict resolution and societal reconstruction, leading to the emergence of new strategic networks and complexes involving governments, non-government organizations, military establishments, and private companies (Duffield 2001a, p. 2).

Particularly important are Duffield’s insights into the incorporation of war and conflict (i.e., trauma, although Duffield does not use this term) into development discourse. Duffield argues that although the destructiveness of conflict was deplored, its wider effects were not construed as wholly negative. Central to this new view of development was the idea that conflict situations present a *positive* opportunity for radical social reconstruction on a grand scale, based on the assumption that the social institutions of a given society had been destroyed by the conflict. It is worth quoting Duffield at length here to provide a sense of the tenor of his argument, and the parallels I seek to draw out between conceptions of trauma and conflict in individual and international development. In Duffield’s (2001a) words:

Although violence can destroy development, a common strand within liberal governance is that is also erodes the cohesion of a society’s culture, customs and traditions. Given that a radicalised development now seeks to transform societies as a whole, including the beliefs and attitudes of the people concerned, this Hobbesian outcome of violence has a certain utility. In ideological terms, it makes the process of transition easier. While the rolling back of development and the deepening of poverty provide the *urgency* to intervene, the destruction of culture furnishes the *opportunity* for aid agencies to establish new and replacement forms of collective identity and social organisation (p. 123, emphasis in original).

The positive utility of “seismic events” such as conflict and warfare in development discourse appears to paradigmatically connect to the positive utility of trauma in psychosocial oncology—a field that is rife with its own militaristic metaphors (Sontag 1990; Penson et al. 2004; Reisfield and Wilson 2004). In both cases, destruction (whether of the self or society as a whole) and the *trauma* it engenders provide both the grounds for intervention and the opportunity to stimulate “development.”

The centrality of trauma to international development discourse becomes particularly evident in the focus on post-*traumatic* stress disorder. Thus, psychosocial interventions are now a key activity of a variety of international aid agencies working in conflict zones, and range from trauma counseling to initiatives to develop life skills and build self-esteem (Pupavac 2001; Fassin and Rechtman 2008). Indeed, as Fassin and Rechtman (2008) have shown, the rise of trauma as an “unassailable moral category” was central to the emergence of humanitarian psychiatry as a legitimate field. Today, psychological care has thus become an integral part of international aid, with psychologists as likely to be sent into emergency situations as doctors. However, although the impetus for intervention is alleviating “post-traumatic stress disorder” rather than facilitating “post-traumatic growth,”^11^ the focus on self-realization and the connections made between emotional literacy and good citizenship (see Pupavac 2001) sound strikingly familiar. In both cases, there is a growing reliance on interventions to stimulate “development” that exploit the opportunities trauma and conflict provide, parceled out through a network of alliances that span the public and private and government and non-government sectors.

## Survivors’ Responses to Dominant Discourses on Cancer and Growth

Despite the questionable ethics of intentionally intervening at moments of “trauma-induced disruption” to stimulate a particular developmental arc, such approaches have received minimal critical scrutiny in the field of psychosocial oncology—or the other disparate arenas where similar ideas appear to be playing out. As Nikolas Rose (1998, pp. 169–190) observes:

In political life, in work, in conjugal and domestic arrangements… and in the apparatuses of medicine and health, human beings are addressed, represented, and acted upon as if they were selves of a particular type: suffused with an individualized subjectivity, motivated by anxieties and aspirations concerning their self-fulfillment, committed to finding their true identities and maximizing their authentic expression in their lifestyles.

Although people treated for cancer undergo diverse transformations that fit poorly into a positive or negative mold marked by either post-traumatic growth or post-traumatic stress disorder (Kahana et al. 2011), in the dominant framework, those who do not experience or desire “growth” are rendered abnormal and pathologized. As Little et al. (2002) note, “The spectrum of socially legitimate responses to the existential state of survivorship is therefore narrowly defined” (p. 176). This narrowing of acceptable responses is illustrated in the quote at the beginning of this essay from Kris Carr, where she emphasizes that survivors are “not imprisoned by the constructs of a label,” but then goes on to do exactly that, suggesting that survivors use their cancer as “a catalyst for creating a better self.”

Cancer survivors themselves are often all too aware of the expectations attached to this identity. For example, Judy Segal (2010), in her newspaper editorial “Cancer isn’t the best thing that ever happened to me,” points to the coercive dimensions of cancer survivorship narratives: “If, as a person with cancer, you violate the code of optimism, or if cancer somehow failed to improve you, you’d better be quiet.” Barbara Ehrenreich (2001, 2009) has similarly criticized the “relentless brightsiding” of cancer and invocations of the “redemptive powers of the disease.” A growing body of qualitative research also speaks to the coercive dimensions of dominant cancer narratives. For example, in their study of thirteen cancer survivors, Little et al. (2002) juxtapose the stories of Bill and Robert, who responded very differently to the experience of colon cancer. While Bill continued to express a sense of restlessness, alienation, anger, and bitterness about his cancer and resulting colostomy, Robert embodied the attributes of the enlightened, gracious, and accepting cancer survivor. As Little et al. note, “Neither Robert’s nor Bill’s response is ‘right’ or ‘wrong’, but the social acceptance of and admiration for one is mirrored by the social distaste for the other” (p. 176).

Breast cancer survivors in Sinding and Gray’s (2005) and Kaiser’s (2008) studies highlight the ways that prevailing discourses on cancer survivorship obscure the ongoing presence of cancer in women’s lives, especially the uncertainty and worry they continue to experience, and the sense of burden placed upon them to stay well. Despite inhabiting this liminal space between the well and the sick, a space that Arthur Frank (1991) has termed “the remission society” and Lochlann Jain (2007) has called “living in prognosis,” survivors spoke of the pressure to hide cancer’s ongoing effects from public view (Sinding and Gray 2005). As Sinding and Gray (2005) observe, “Survivors are required to act as if cancer is over, and at the same time they are required to act to prevent recurrence. The first requirement rejects or pathologizes an ongoing sense of vulnerability to cancer; the latter is premised on it” (p. 157).

As I have previously documented, cancer survivors are often highly susceptible to messages about the role of lifestyle in tertiary cancer prevention, and tend to see this as a way of exerting some individual control over the possibility of disease recurrence (Bell 2010). However, this need for vigilance around lifestyle invariably leads to a sense of guilt when survivors experience a lapse in will power or fail to live up to what they see as a healthy lifestyle (Bell 2010). As a woman in Sinding and Gray’s (2005) study commented, “Every time I put something in my mouth I’m aware and vigilant—if I have (fast food), I need lots of salads after. I keep a little tally of good things, bad things…” (p. 152). Similarly, in Broom and Tovey’s (2008) study of cancer patients’ experiences of CAM therapies, they found that therapeutic regimes often created a form of governance of the self whereby patients “…felt bad if they slept in, ‘missed an enema’ or had negative thoughts” (p. 1655). In the context of an actual cancer recurrence, this sense of self-blame and responsibility is likely to be dramatically intensified.

Should the fear that surrounds cancer, still the most culturally loaded of all diseases despite some heavy competition from HIV/AIDS, be used to encourage survivors to fit into a particular vision of what the self should be and aspire to? While undoubtedly well intended and ostensibly designed with the best interests of the patient in mind, prevailing interventions to promote physical and psychological health serve to naturalize a problematic set of assumptions about trauma and the self, while simultaneously assigning the responsibility for regaining and maintaining health to the patient. While there are those for whom such discourses are empowering, providing a means of gaining something positive out of what otherwise seems like a senseless and destructive illness, in light of the groundswell of critique from survivors themselves their underside demands further attention.

## Conclusions

Popular notions regarding the transformative dimensions of the cancer experience clearly infuse oncological discourses on cancer survivorship, crystallizing most explicitly in concepts such as the “teachable moment” and “post-traumatic growth”—which suggest that cancer diagnosis motivates survivors to transform not only their lifestyles but their psychological selves as well. Increasingly, however, these concepts have become not merely *descriptions* of the experience of cancer survivorship but *prescriptions* for how survivors should conduct themselves. Indeed, I have argued that the trauma a cancer diagnosis is seen to entail is increasingly understood to have positive, instrumental value. In shattering closely held illusions about the self, it is deemed to present an invaluable opportunity to establish new and replacement forms of identity in those patients who have not spontaneously re-evaluated their lifestyles and selves.

I have suggested that these assumptions are not confined to the field of psychosocial oncology; indeed, as previously outlined, the concepts of the “teachable moment” and “posttraumatic growth” have a wide degree of currency in the fields of health promotion and health psychology. Moreover, by paradigmatically linking together individuals and collectivities, these conceptions of “trauma” and “development” have implications for an array of disparate contexts. They speak to a larger ideological shift in contemporary forms of governance, particularly a growing cynicism about whether development (physical, psychological, or social) will occur “naturally” if individuals and societies are left to their own devices, and an attendant appetite for intervention. Trauma and conflict—within the self or external to it—are central to this new strain of interventionism, seen to create a kind of *tabula rasa* that allows selves and societies to be fundamentally remade.

Prevailing frameworks and interventions rely on a set of assumptions about the nature of selfhood (and statehood) that have become thoroughly naturalized today. These assumptions presuppose the desirability of the autonomous, responsible subject/citizen obliged to make his or her life meaningful through acts of choice that maximize emotional, physical, and economic health (Rose 1998; Miller and Rose 2008) and attendant social and political systems aligned with the rationalities of liberal modernity (Escobar 1995; Duffield 2001a, 2001b). However, the hegemonic dimensions of this discourse and its codification in the various subfields of psychosocial oncology deserve critical scrutiny in light of the narrow range of acceptable responses to cancer they imply.

## Footnotes

1 Much of the discussion centered on the need for “objective” measurements of benefit finding and post-traumatic growth. The general tenor of the talks and following discussion was that the self-assessments of cancer survivors could not be trusted as they may falsely think they have experienced growth because of the “tyranny of positive thinking.” Indeed, researchers working in this area increasingly stress the need to distinguish real growth from “pseudo-growth” (e.g., Lechner and Antoni 2004) because of similar concerns. Yet, although such discussions would appear to mount a challenge to the validity of the construct of post-traumatic growth itself, instead it is the survivors themselves who are challenged: the construct is “real” but survivors’ reports are untrustworthy. Survivors, it appears, are “damned if they do and damned if they don’t.” Interventions are necessary to facilitate post-traumatic growth, but survivors’ reports on whether growth has been achieved are deemed faulty.

2 It is also arguable whether these discourses apply equally to elderly people diagnosed with cancer, where the tragedy of cancer is seen to be less acute, coming as it does at the end of a long life. Sinding and Wiernikowski (2008), in their study of older women with cancer, show that women did not see cancer as intrinsically traumatic and transformative. Rather, it was seen as an inevitable part of getting older that in some cases receded in light of other chronic diseases.

3 In the words of Humpel, Magee, and Jones (2007, p. 622), “Positive lifestyle changes are more likely to occur if the cancer survivor believes that these changes will improve their health and prevent cancer reoccurrence.” There is clearly a sales pitch that occurs, whereby survivors need to be convinced (in the face of inconclusive evidence) of the tangible benefits of making this change (Bell and Ristovski-Slijepcevic under review). As Balshem (1993) has pointed out, many of the “myths” about cancer that health educators are expected to dispel are actually valid and logical interpretations of the existing evidence.

4 However, this assumption is not necessarily accurate. As pointed out in note #2, not all of those who experience cancer find the experience equally traumatic. This, of course, speaks to some of the limitations of existing ways of thinking about trauma, a category which serves to universalize and normalize experience and erase difference (see Young 1995; Pupavac 2001; Fassin and Rechtman 2008).

5 The cover of the first book on this topic actually depicts a cloud with a silver lining.

6 As noted above, this framing is very similar to the ways that PTSD is represented. For example, one website on PTSD describes it in the following terms: “sometimes the trauma is so overwhelming that you find that you can’t move on. You feel stuck…. If you went through a traumatic experience and are having trouble getting back to your regular life…you may be suffering from post-traumatic stress disorder” (Smith and Segal 2011).

7 Although psychosocial oncology (also known as psycho-oncology) emerged in the late 1960s/early 1970s, and was the focus of a specialty journal (*Journal of Psychosocial Oncology*) from the mid 1980s; arguably, it was not until the early 1990s that it was recognized as a mainstream subspecialty (see Holland 1992).

8 Such alliances are a good example of the public–private–NGO partnerships that have arisen over the past two decades around the issue of “cancer survivorship” (see Klawiter 2008), where a growing amount of survivorship “care” is managed by dispersed and decentralized community organizations.

9 Indeed, evidence suggests that identifying with the “survivor” identity leads to taking a more “active” approach to dealing with cancer (Deimling et al. 2007).

10 Interestingly, Cancer Support Community now uses a modified (and less loaded) definition of “patient active” on their website: “People with cancer who actively participate in their fight for recovery along with their physicians and healthcare professionals will improve the quality of their lives and may enhance the possibility of their recovery,” although the original version of the concept is still evident in many of their materials.

11 Interestingly, although discussions of “post-traumatic growth” and “post-traumatic stress” occur in both bodies of literature, the concepts are not equally weighted: post-traumatic growth dominates discussions of cancer and post-traumatic stress dominates discussions of the effects of war and conflict situations. Invoking post-traumatic growth to speak of the aftermath of civil war seems inherently problematic, because of the concept’s implications (explicit or not) that the trauma is, at the end of the day, a good thing. These implications are very much a part of dominant cancer narratives (Segal 2010). This speaks to the moral economy of trauma and differing valuations placed upon it depending on where the responsibility for the trauma is seen to lie (Fassin and Rechtman 2008).
